# Ionic Liquids as Delaminating Agents of Layered Double Hydroxide during In-Situ Synthesis of Poly (Butylene Adipate-*co*-Terephthalate) Nanocomposites

**DOI:** 10.3390/nano9040618

**Published:** 2019-04-16

**Authors:** Hynek Beneš, Jana Kredatusová, Jakub Peter, Sébastien Livi, Sonia Bujok, Ewa Pavlova, Jiří Hodan, Sabina Abbrent, Magdalena Konefał, Petra Ecorchard

**Affiliations:** 1 Institute of Macromolecular Chemistry of the Czech Academy of Sciences, Heyrovského nám. 2, 162 06 Prague 6, Czech Republic; jana.kredatusova@email.cz (J.K.); peter@imc.cas.cz (J.P.); bujok@imc.cas.cz (S.B.); pavlova@imc.cas.cz (E.P.); hodan@imc.cas.cz (J.H.); abbrent@imc.cas.cz (S.A.); magdalenakonefal@imc.cas.cz (M.K.); 2 Université de Lyon, CNRS, UMR 5223, Ingénierie des Matériaux Polymères, INSA Lyon, F-69621 Villeurbanne, France; sebastien.livi@insa-lyon.fr; 3 Institute of Inorganic Chemistry of the Czech Academy of Sciences, Husinec-Řež č.p. 1001, 25068 Řež, Czech Republic; ecorchard@iic.cas.cz

**Keywords:** poly (butylene adipate-*co*-terephthalate), ionic liquid, layered double hydroxide, in-situ polymerization, nanocomposite, permeability, biodegradable polymer

## Abstract

Currently, highly demanded biodegradable or bio-sourced plastics exhibit inherent drawbacks due to their limited processability and end-use properties (barrier, mechanical, etc.). To overcome all of these shortcomings, the incorporation of lamellar inorganic particles, such as layered double hydroxides (LDH) seems to be appropriate. However, LDH delamination and homogenous dispersion in a polymer matrix without use of harmful solvents, remains a challenging issue, which explains why LDH-based polymer nanocomposites have not been scaled-up yet. In this work, LDH with intercalated ionic liquid (IL) anions were synthesized by a direct co-precipitation method in the presence of phosphonium IL and subsequently used as functional nanofillers for in-situ preparation of poly (butylene adipate-*co*-terephthalate) (PBAT) nanocomposites. The intercalated IL-anions promoted LDH swelling in monomers and LDH delamination during the course of in-situ polycondensation, which led to the production of PBAT/LDH nanocomposites with intercalated and exfoliated morphology containing well-dispersed LDH nanoplatelets. The prepared nanocomposite films showed improved water vapor permeability and mechanical properties and slightly increased crystallization degree and therefore can be considered excellent candidates for food packaging applications.

## 1. Introduction

Currently, engineering plastics are designed as sustainable materials with an adjustable lifetime in order to minimize their impact on the environment. The challenging issue is thus to prepare advanced plastics either with (i) increased durability or (ii) easy and fast degradability by means of abiotic factors (humidity, temperature, pH, etc.) and/or biological (microbial) attack. The first are hi-performance materials with a long service life, while the latter are often designed as biodegradable polymers for packaging with a typical life-time shorter than one year.

Poly (butylene adipate-*co*-terephthalate) (PBAT) is a typical representative of aliphatic-aromatic copolyesters, which provide balance between biodegradability of aliphatic segments and thermo-mechanical properties of aromatic units. PBAT is a flexible material with high elongation at break and thus suitable for coatings and film applications [1]. However, its low crystallization degree results in insufficient stiffness and modulus. Together with low water vapor barrier properties, these are the main drawbacks limiting the PBAT usage for food packaging [2,3]. A possibility to overcome the PBAT shortcomings is to incorporate lamellar inorganic particles, such as cationic clays (talc and montmorillonites [2,4,5,6,7]), anionic clays such as layered double hydroxides (LDH) [8], and carbon compounds such as graphite or graphene-based nanoplatelets [9,10] in the PBAT matrix.

LDH are particularly advantageous due to their mild and efficient synthesis, chemical structure versatility and anionic exchange ability, which enables tuning of the LDH surface interactions for targeted nanocomposite fabrication [11]. The LDH or hydrotalcite-like compounds are represented by the general formula [M^2+^_1−x_M^3+^_x_(OH)_2_]^x+^(A^n−^)_x/n_·mH_2_O, where M^2+^ and M^3+^ are divalent (Ca^2+^, Mg^2+^, Fe^2+^, Zn^2+^, Cu^2+^, Co^2+^) and trivalent (Fe^3+^, Al^3+^, Bi^3+^, In^3+^, La^3+^, Ga^3+^) layer cations, respectively, and A^n−^ is the exchangeable inorganic anion (Cl^−^, OH^−^, CO_3_^2−^, SO_4_^2−^, NO_3_^−^) in the interlayer space with x being the relative substitution rate generally ranging between 0.20 < x < 0.33 resulting in M^2+^/M^3+^ ratios of 2/1–4/1 [12,13].

The layered inorganic compounds can act as nano-sized fillers in a polymer matrix providing high reinforcing and barrier effects only when their structure is delaminated [14]. Additionally, the resulting nanocomposites with an intercalated or, more preferentially, exfoliated structure must exhibit strong interfacial interactions between the nanofiller surface and the polymer matrix. In our last paper, we have demonstrated that phosphonium ionic liquids (IL) can act as surfactants to LDH, which disperse the LDH nanoparticles in the PBAT matrix during the melt intercalation [8]. The improved compatibility between LDH and the PBAT matrix was shown to result in increased mechanical and water barrier properties of the PBAT/LDH nanocomposites as compared to neat PBAT. However, melt blending did not allow for full exfoliating of the LDH structure and thus only partially intercalated PBAT/LDH nanocomposites were prepared.

There exist several strategies in how to delaminate the multi-lamellar structure of LDH and to prepare fully exfoliated PBAT/LDH nanocomposites: Solvent intercalation, melt blending, and in-situ polymerization. During solvent intercalation, LDH is exfoliated using a solvent (e.g., chloroform) in which PBAT is soluble [15]. The method is not suitable for preparation of bulk PBAT/LDH nanocomposites since a volatile organic solvent is used and must be subsequently removed. The melt blending involves dispersing of LDH in PBAT in the molten state using a strong shear force [16,17]. This straightforward technique does not require any solvent and is most commonly used for the dispersion of cationic clays (smectitic clay minerals, e.g., montmorillonites, MMT) in PBAT [2], while its application for exfoliation of bulky anionic clays (LDH) was found more difficult due to their high charge density [18]. This method, however, does not permit full exfoliation of all LDH particles, and often a mixture of mono- and multi-lamellar sheets is received [8]. The in-situ polymerization consists of LDH swelling in the mixture of monomers, followed by polycondensation during which the chain growth in the interlayer space accelerates LDH delamination [19,20]. The exfoliation process of the swollen LDH layers is further supported by external forces via ultrasonic treatment or mechanical high shear mixing [18]. A suitable organic modification of LDH by anionic exchange is often necessary in order to facilitate monomer diffusion into the LDH interlayer space and at the same time initiate or catalyze the polymerization process. It has been shown that IL are ideal candidates for this kind of LDH treatment.

Ionic liquids (IL) are organic salts with low melting temperature (<100 °C) exhibiting several unique properties, e.g., excellent thermal and chemical stability, low vapor pressure and no flammability, which makes them suitable for instance as additives for polymers [21]. Moreover, ILs offer broad range of organic anions, which are not always commercially available as simple salts. Finally, the prices of ILs and simple salts are comparable. Therefore, their use for LDH (or in general inorganic filler) modification is convenient and furthermore mild conditions can be applied for filler modification. We have shown that organic anions of IL introduced into the LDH interlayer space are able to delaminate the LDH compounds and simultaneously initiate in-situ polymerization of ε-caprolactone [22].

In this paper, we use a similar approach to investigate in-situ prepared PBAT/LDH nanocomposites. First, Mg-Al LDH modified with different IL-anions are synthesized using direct co-precipitation. Contrary to the previously used anion exchange reaction producing mainly IL-surface-modified LDH [8], the co-precipitation technique enables it to reach higher content of intercalated IL-anions and thus to promote LDH exfoliation during polymerization. In the next step, PBAT nanocomposites are prepared in the presence of modified LDH via in-situ polycondensation. Finally, the influence of 1.5 and 5 wt % addition of IL-modified LDH on morphology, water/gas permeability, thermal, and mechanical properties of the PBAT/LDH nanocomposites is determined.

## 2. Materials and Methods

Trihexyltetradecylphosphonium bis (2,4,4-trimethylpentyl) phosphinate (IL-phosphinate), trihexyltetradecylphosphonium decanoate (IL-decanoate), and trihexyltetradecylphosphonium bis (2-ethylhexyl) phosphate (IL-phosphate) (see Table 1) were provided by IoLiTec (Heilbronn, Germany). MgCl_2_·6H_2_O, AlCl_3_·9H_2_O, NaOH, NH_4_OH, NaCl, THF (p.a.), CHCl_3_ (p.a.), ethanol (p.a.), and methanol (p.a.) were supplied by Lach-Ner (Neratovice, Czech Republic). All chemicals necessary for synthesis of LDH were used as received without further purification.

### 2.1. Preparation of LDH with Intercalated IL-Anions

Direct co-precipitation of metal salts in the presence of different ILs was used for the preparation of the LDH with intercalated IL-anions. Calculations were made for 2 g of LDH; ratio of IL/LDH was 1.5/1 (calculated according to anionic exchange capacity of LDH 3.35 meq/g). In a three necked flask, IL was first dissolved in a suitable organic solvent and subsequently an aqueous solution (25 mL) of MgCl_2_·6H_2_O and AlCl_3_·9H_2_O (Mg/Al molar ratio of 2/1) was added dropwise. The exact weights were as follows: 7.8 g (0.01 mol) of IL-phospinate was dissolved in 50 mL of THF and the solution of MgCl_2_·6H_2_O (4.3 g, 0.02 mol) and AlCl_3_·9H_2_O (2.6 g, 0.01 mol) in deionized water (25 mL) was added; 6.7 g (0.01 mol) of IL-decanoate was dissolved in 40 mL of CHCl_3_ and the solution of MgCl_2_·6H_2_O (4.3 g, 0.02 mol) and AlCl_3_·9H_2_O (2.6 g, 0.01 mol) in deionized water (25 mL) was added; 8.1 g (0.01 mol) of IL-phosphate was dissolved in 40 mL of ethanol and the solution of MgCl_2_·6H_2_O (4.3 g, 0.02 mol) and AlCl_3_·9H_2_O (2.6 g, 0.01 mol) in deionized water (25 mL) was added. The molar IL/Al ratio of 1/1 was always used. In the case of IL-decanoate, we first tried to apply mixtures of miscible solvents but IL-decanoate remained dissolved, having no tendency to bond anions onto LDH and stack layers together. LDH with no significant IL-decanoate intercalation were obtained. Therefore, an immiscible CHCl_3_/water mixture together with vigorous stirring led to swelling of the created LDH and its subsequent intercalation. The pH was kept constant at pH = 10 with the addition of NH_4_OH. The resulting slurry was then aged at 60 °C for 24 h. Finally, the LDH precipitate was filtered, washed with a mixture of water and organic solvent, and dried at 80 °C for 48 h under vacuum. All steps were conducted under nitrogen atmosphere to avoid contamination by carbonate. Powders of three LDH modified with IL-phosphinate, IL-decanoate, and IL-phosphate were prepared and denoted as LDH-phosphinate, LDH-decanoate, and LDH-phosphate, respectively.

As reference material, Cl^−^-intercalated Mg-Al LDH (pristine LDH) was prepared by the co-precipitation method adapted from [23]. Briefly, 150 mL of an aqueous solution of NaOH (35 mmol) and NaCl (34 mmol) was slowly titrated with an ethanol-water solution of MgCl_2_·6H_2_O and AlCl_3_·9H_2_O (Mg/Al molar ratio of 2/1, cations concentration 0.375 mol/L) at 80 °C under nitrogen atmosphere. The formed precipitate was then aged for 1 h. The final sludge was filtrated on a Büchner funnel, washed with deionized water, and dried at 80 °C for 12 h to obtain the white powder of pristine LDH.

### 2.2. Preparation of PBAT/LDH Nanocomposites

Poly (butylene adipate-*co*-terephthalate) (PBAT)/LDH nanocomposites were prepared via an in-situ polycondensation of 1,4-butanediol (BD, >99%, Sigma-Aldrich, Saint Louis, MO, USA), dimethyl terephthalate (DMT, >99%, Sigma-Aldrich, Saint Louis, MO, USA), and dimethyl adipate (DMA, >99%, Sigma-Aldrich, Saint Louis, MO, USA) and catalyzed by 0.3% tetra-n-butyl orthotitanate (>97%, Sigma-Aldrich, Saint Louis, MO, USA) according to the two-step procedure adapted from [24]. Prior to the first step, a given amount of LDH powder was freshly dried (80 °C under vacuum) and then dispersed in BD using an ultrasound bath for 10 min. In the first step, BD (62 mmol, 20% molar excess) with dispersed LDH, DMT (21 mmol) and catalyst (0.15 mmol) were charged into a 100 mL three-neck flask equipped with a magnetic stirrer, a nitrogen inlet and a distillation column. The reaction took place under nitrogen flow at 190 °C for ca 1 h until methanol was completely distilled out. In the second step, DMA (31 mmol) was added into the reaction flask. As soon as methanol distillation was completed, the temperature of reaction mixture was increased to 220 °C and the pressure was gradually decreased under a final reduced pressure lower than 25 Pa for 2 h. The prepared highly viscous melt of PBAT was then cooled down to room temperature, dissolved in chloroform, precipitated into methanol, and dried at 60 °C under vacuum. The obtained nanocomposite was analyzed using ^1^H NMR (Figure S1) and SEC (PS standards) showing the ratio of butylene adipate (BA)/butylene terephthalate (BT) units of 59/41 (theor. 60/40) and M_w_ of ca. 45,000 g/mol (M_w_/M_n_ = 1.6). The LDH content in PBAT nanocomposites was 1.5 and 5.0 wt %. The same two-step procedure was used for the synthesis of reference neat PBAT.

For further characterization and testing, the PBAT and PBAT/LDH nanocomposites were prepared as films (the thickness of 200 μm) using compression molding (PTFE molds) at 130 °C with a compression force of 50 kN for 2 min.

### 2.3. Characterizations

Infrared (FTIR) spectra of the LDH samples were measured using the attenuated total reflectance (ATR) technique on a spectrometer Spectrum 100T FT-IR (PerkinElmer, Waltham, MA, USA) with a deuterated triglycine sulfate (DTGS) detector fitted with a Universal ATR accessory with a diamond/ZnSe crystal. All spectra were recorded in the wavenumber range of 650–4000 cm^−1^ at 16 scans per spectrum and 4 cm^−1^ resolution.

^1^H NMR spectra (600 MHz) of the prepared PBAT and PBAT/LDH nanocomposites were obtained using a Bruker Avance III 600 MHz NMR spectrometer with CDCl_3_ as the solvent at 25 °C. The chemical shifts are relative to TMS using hexamethyldisiloxane (HMDSO, 0.05 ppm from TMS) as the internal standard.

X-Ray diffraction (XRD) patterns were obtained using a high-resolution diffractometer explorer (GNR Analytical Instruments, Novara, Italy) equipped with a one-dimensional silicon strip detector Mythen 1K (Dectris, Baden, Switzerland). The CuKα radiation (wavelength λ = 1.54 Å) was produced by a sealed X-ray tube operated at 40 kV and 30 mA and monochromatized with Ni foil (β filter). The measurements were performed in Bragg-Brentano geometry in the range of *2θ* = 2–70° with a step 0.2°. The exposure time at each step was 10 s.

Thermogravimetric analysis (TGA) of the LDH samples and the PBAT/LDH nanocomposite films was performed using a Pyris 1 TGA (PerkinElmer, Waltham, MA, USA) in a temperature range from 35 to 750 °C at a rate of 10 °C/min; the purge gas flow rate was fixed at 25 mL/min of nitrogen. Temperature of 5% weight loss (*T_d_*_5%_) was evaluated for the nanocomposite samples. Standard deviation of TGA measurement was under 5%.

Transmission electron microscopy (TEM) was performed on a Tecnai G2 Spirit Twin 12 microscope (FEI, BrNo, Czech Republic) in the bright field mode at the acceleration voltage of 120 kV. The PBAT/LDH nanocomposite films were cut into ultrathin sections (approximately 60 nm thick) by a Cryo-ultramicrotomy (Ultracut UCT, Leica, Wetzlar, Germany) using sample and knife temperatures of −80 °C and −40 °C, respectively.

Oxygen, carbon dioxide and water vapor transport properties of the PBAT/LDH nanocomposite films were examined by time-lag permeation method [25]. Each sample was inserted into a membrane cell which was then placed into a permeation apparatus and exposed to high vacuum (10^−4^ mbar) and a temperature of 45 °C for 12 h. After evacuation the temperature was set to 30 °C. Feed pressure *p_i_* was 1.5 Bar. The permeability coefficient *P* was determined from the increase of the permeate pressure Δ*p*_p_ per time interval Δ*t* in a calibrated volume *V*_p_ of the product part during the steady state of permeation. For the calculation of the permeability coefficient, the following formula was used:

*P* = (Δ*p*_p_/Δ*t*)·[*V*_p_*l/*(*Ap_i_*)]·[1/(*RT*)] (1)

where *l* is the membrane thickness, *p_i_* feed pressure, *A* the area, *T* the temperature, and *R* the gas constant. Two or three specimens of each PBAT/LDH nanocomposite films were measured and the average reported. Relative standard deviations (SD) of Δ*p*_p_ and Δ*t* were lower than 0.3% (given by the 10 mbar MKS Barratron pressure transducer precision). Relative SD of membrane thickness measurement was 1%, relative SD of calibrated volume was lower than 0.5%, and relative SD of feed pressure was 0.3%. Therefore *P* values had the relative SD 2.4%. Gas diffusivities were estimated from the time-lag data, using the relation:

*D* = *l*^2^/(6*θ*) (2)

where *l* is the film thickness and *θ* is the time lag. Relative SD of diffusion coefficients was 4%. Apparent solubility coefficients were calculated using the following equation:

*S* = *P*/*D* (3)

The overall ideal selectivity (α*_ij_*) of a polymer membrane for a pair of gases *i* and *j* is commonly expressed by the following relation:

α*_ij_* = *P_i_*/*P_j_* = (*S_i_*/*S_j_*)·(*D_i_*/*D_j_*) (4)

where *P_i_* and *P_j_* are pure gas permeabilities, *D_i_*/*D_j_* is the diffusion selectivity, and *S_i_*/*S_j_* is the solubility selectivity.

The thermal behaviors of the PBAT/LDH nanocomposite films were investigated using a DSC Q2000 calorimeter (TA Instruments, New Castle, DE, USA) with nitrogen purge gas (50 mL/min). The instrument was calibrated for temperature and heat flow using indium as a standard. Samples of about 10 mg were encapsulated into aluminum pans. Differential scanning calorimetry (DSC) was performed with a heating-cooling-heating cycle from −90 °C to 200 °C at 10 °C/min. Before and after the ramps, a two minute isothermal plateau was inserted. The glass transition temperature (*T_g_*), melting temperature (*T_m_*), and melting enthalpy (Δ*H_m_*) were determined from the second heating scans. The crystallinity (*X_c_*) of PBAT and PBAT nanocomposites was calculated using the following expression:

*X_c_* = 100·Δ*H_m_*/[Δ*H_m_^o^*·(1 − *w_f_*)] (5)

Δ*H_m_* is the specific melting enthalpy of the sample, Δ*H_m_^o^* is the melting enthalpy of the 100% crystalline PBAT (114 J/g [26,27]), and *w_f_* is the weight fraction of LDH filler.

The tensile tests on the PBAT/LDH nanocomposite films were conducted at ambient temperature using an Instron model 6025/5800R (Instron Limited, Norwood, MA, USA) equipped with a 100 N load cell at room temperature with a cross-head speed of 50 mm min^−1^. Dumbbell-shaped specimens (ISO 527-3/5, half size) were used having the length of 60 mm, length and width of the narrow part: 16.5 and 3 mm, resp. and average thickness of ca 0.2 mm. Five specimens of each PBAT/LDH nanocomposite films were measured and the average reported.

## 3. Results and Discussion

### 3.1. Synthesis of Layered Double Hydroxide with Intercalated Ionic Liquid Anions

#### 3.1.1. FTIR Spectra

**Figure 1:**
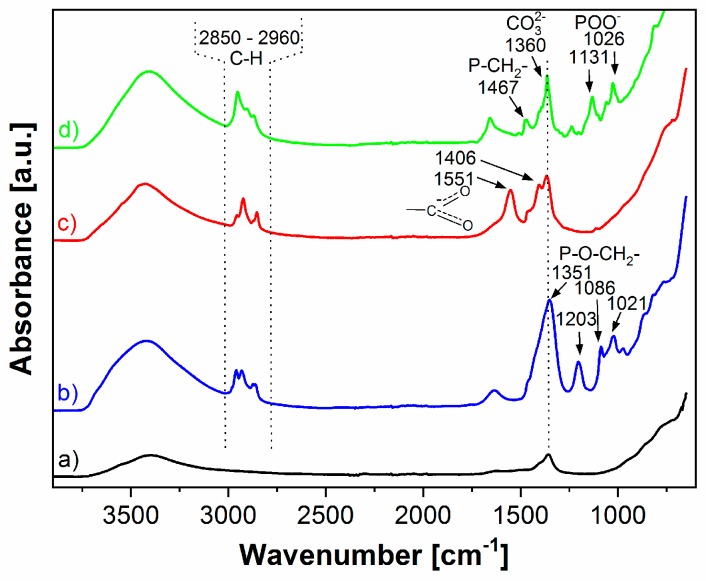
FTIR spectra of (**a**) pristine LDH, (**b**) LDH-phosphate, (**c**) LDH-decanoate, and (**d**) LDH-phosphinate.

The FTIR spectra of modified LDH (Figure 1) confirmed a significant content of organic phase due to the presence of IL-anions. A strong band of methylene deformation (PCH_2_-) of phosphonium cations at 1466 cm^−1^, typical for all the used phosphonium ILs (see FTIR spectra in the supplementary material, Figure S2) [28], was absent in all of the modified LDH (Figure 1b–d). The presence of phosphonium cations in all prepared IL-modified LDH was not detected. Therefore, the content of ILs adsorbed on the LDH surface can be neglected. It can be concluded that direct synthesis has led to a successful intercalation of IL-anions and the prepared LDH contains an organic part composed uniquely of the IL-anions.

The FTIR spectrum of LDH-decanoate (Figure 1c) exhibited strong asymmetric and symmetric carboxylate anion stretching bands at 1551 cm^−1^ and 1406 cm^−1^, respectively [29,30], proving the presence of IL-decanoate anions in the LDH-decanoate.

The FTIR spectrum of LDH-phosphinate (Figure 1d) clearly evidences the presence of phosphinate anions as the band at 1467 cm^−1^ can be assigned to methylene deformation of PCH_2_– [29] and the peaks at 1131 cm^−1^ and 1026 cm^−1^ correspond to asymmetric and symmetric (P = O) O-stretching [30].

The strong bands at 1351 cm^−1^ and the medium bands at 1203 cm^−1^, 1086 cm^−1^, and 1021 cm^−1^ in the FTIR spectrum of LDH-phosphate (Figure 1b) were assigned to P–O–CH_2_– stretching [29], which evidences the presence of IL-phosphate anions.

Besides the IL-anions, the presence of carbonate anions in all of the modified LDH (as well as in the pristine LDH) was confirmed by the FTIR band at ca 1360 cm^−1^ (Figure 1) [31]. The direct synthesis was found to be highly efficient and led to preparation of LDH with a much higher content of organic anions (see the TGA results bellow) compared to the regeneration method published in our last study [22]. However, the presence of carbonate anions cannot be completely avoided, probably due to very high affinity of CO_3_^2−^ to LDH [32]. The FTIR spectra of all LDH also contained absorption band at ca 1650 cm^−1^ typical for H–O–H deformation vibration of the interlayer water [11,33].

#### 3.1.2. XRD Patterns

**Figure 2:**
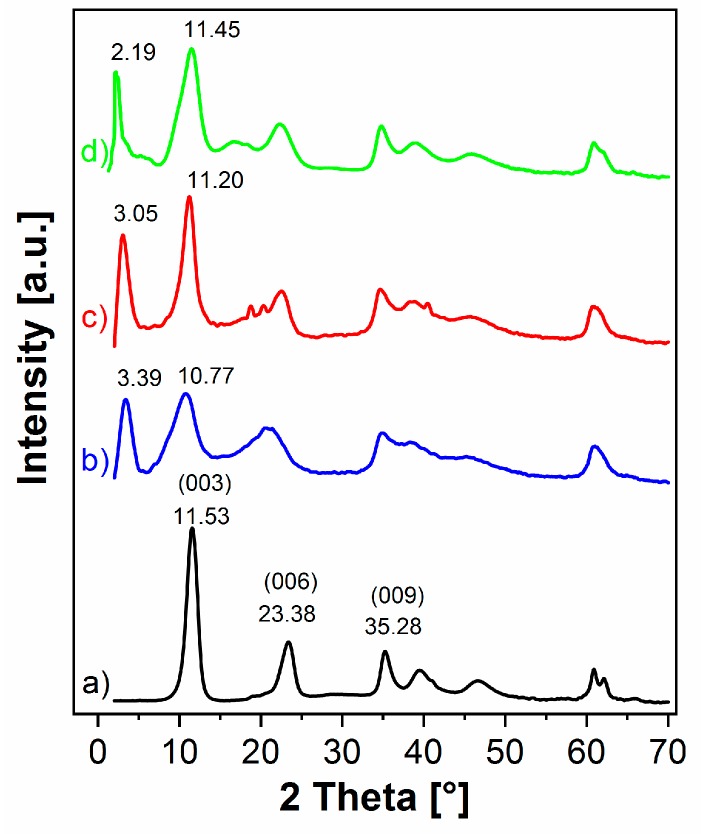
XRD patterns of (**a**) pristine LDH, (**b**) LDH-phosphate, (**c**) LDH-decanoate, and (**d**) LDH-phosphinate.

The XRD measurements enabled us to indicate whether the IL-anions were intercalated into the basal spacing of LDH. The XRD pattern of pristine LDH (Figure 2a) showed the formation of a single-phase, well-ordered crystalline-layered structure. The peaks located at 2*θ* = 11.53°, 23.38°, and 35.28° were attributed to the diffraction by (003), (006), and (009) planes, respectively. The (003) reflection of pristine LDH corresponded to the basal spacing value of 0.77 nm, which is typical for unmodified Mg-Al LDH [34,35,36].

The XRD patterns of the LDH modified with ILs demonstrated that the layered structure of the LDH was preserved during the one step LDH synthesis via co-precipitation. The presence of (003) reflection in the lower 2*θ* range—3.39°, 3.05°, and 2.19° for LDH-phosphate, LDH-decanoate, and LDH-phosphinate, respectively (Figure 2), denotes the basal spacing expansion to 2.6 nm, 2.9 nm, and 4.0 nm, respectively. This proves that the intercalation of IL anions, which size is larger than Cl^−^, into the interlayer of LDH occurs. The obtained basal spacing expresses the sum of the thickness of one brucite-like octahedral layer and interlayer spacing. The later can be affected by the size and orientation of interlayer anion [36]. Assuming the thickness of brucite-like layer of ca 0.48 nm [37], then the interlayer spacing for LDH-phosphate, LDH-decanoate, and LDH-phosphinate expanded from 0.29 nm (for the pristine LDH) to approximately 2.1 nm, 2.4 nm, and 3.5 nm, respectively. The position and shape of broad XRD peaks at 10.77°, 11.20°, and 11.45° present for LDH-phosphate, LDH-decanoate, and LDH-phosphinate, respectively, suggest overlapping of two peaks. One of them could be ascribed to the shifted (006) reflection of intercalated LDH phase, while the second suggests possible generation of the second phase with smaller basal spacing. This is also supported by the shape of the peaks in 2*θ* = 20°–23° present in all modified samples, which are also much broader than corresponding peaks in pristine LDH sample. The formation of the second phase could be caused by the different orientation of IL-anions inside the LDH interlayer spacing as was described by other authors [36] or by the generation of CO_3_^2−^-intercalated Mg-Al LDH (as supported by FTIR, Figure 1).

#### 3.1.3. Thermogravimetric Analysis

**Figure 3:**
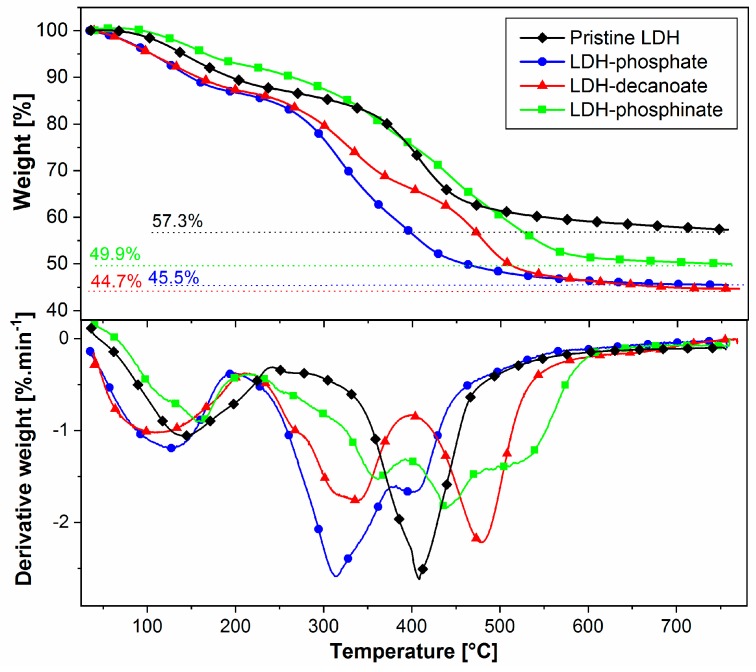
Thermogravimetric and derivative weight curves of pristine LDH, LDH-phosphate, LDH-decanoate, and LDH-phosphinate. The heating ramp was performed at 10 K·min^−1^ under N_2_ atmosphere.

Thermogravimetric (TG) and derivative weight TG curves of all LDH samples display an initial weight loss (8–13 wt %) between 50 °C and 250 °C (Figure 3) due to the release of physisorbed and interlayer water [17,22]. The TG curve of pristine LDH exhibits the removal of an interlayer carbonate anion and dehydroxylation of –OH groups between 250 °C and 500 °C (weight loss of ca 30 wt %) [38]. Moreover, TGA results of the modified LDH clearly show the degradation of intercalated IL-anions in the range of 250–500 °C. Significant weight loss of ca 42 wt % was observed in all modified LDH. Minimal contents of intercalated IL-anions in the modified LDH were estimated from the mass loss differences between the pristine LDH and the modified LDH to 11.8 wt % (LDH-phosphate), 12.6 wt % (LDH-decanoate), and 7.4 wt % (LDH-phosphinate, Figure 3). Unfortunately, the exact amount of intercalated IL-anions cannot be determined from TGA, because the IL-anion degradation, the interlayer CO_3_^2−^ decomposition, and dehydroxylation of the metal hydroxides proceed simultaneously.

### 3.2. Characterization of PBAT/LDH Nanocomposites

#### 3.2.1. Morphology

**Figure 4:**
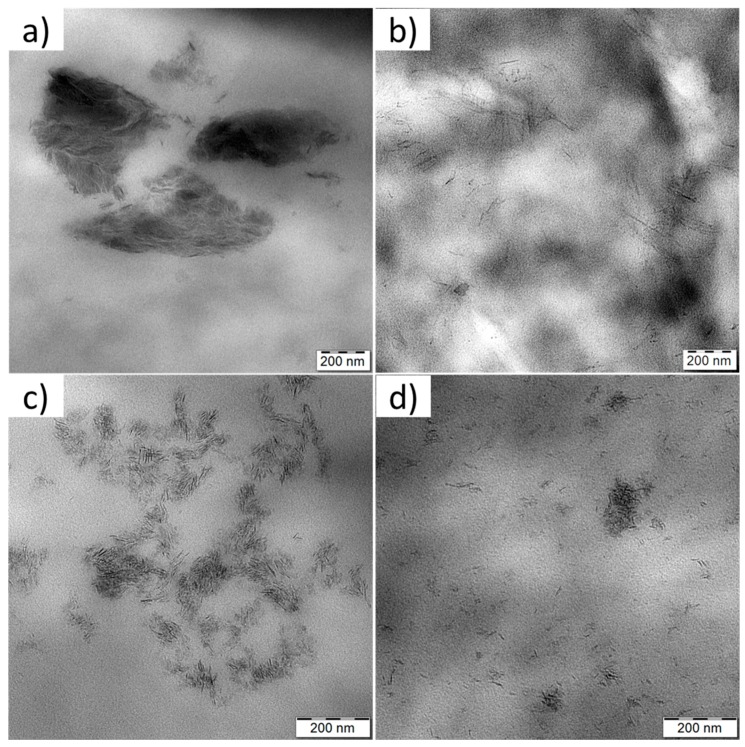
TEM images of PBAT nanocomposites containing 5 wt % of (**a**) pristine LDH, (**b**) LDH-phosphate, (**c**) LDH-decanoate, and (**d**) LDH-phosphinate.

**Figure 5:**
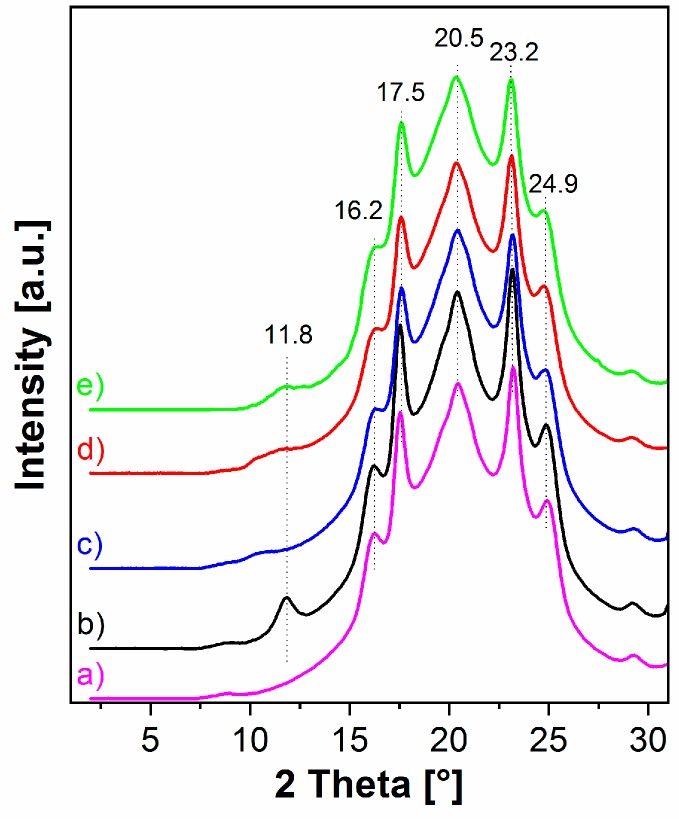
XRD patterns of (**a**) neat PBAT and PBAT nanocomposites with 5 wt % of (**b**) pristine LDH, (**c**) LDH-phosphate, (**d**) LDH-decanoate, and (**e**) LDH-phosphinate.

TEM images revealed that the type of LDH modification had strongly affected the final morphology of PBAT nanocomposites with 5 wt % of LDH filler (Figure 4). The non-modified LDH showed poor dispersion in the PBAT precursors with a low tendency to swell during the in-situ polymerization, which resulted in a composite containing large stacks of LDH (Figure 4a). Contrary to that, the successful IL-anion intercalation promoted LDH dispersion and swelling in the mixture of monomers resulting in the intercalated and exfoliated morphology of the final PBAT nanocomposites. Delamination of the modified LDH during the in-situ polymerization proceeded more easily due to weaker forces between organic IL-anions and the LDH layers as compared to small inorganic anions that hold the LDH layers together [39,40]. However, a fully exfoliated morphology of PBAT nanocomposites was not reached because a certain amount of CO_3_^2−^-intercalated into Mg-Al LDH was always present as a contaminant. The PBAT nanocomposites containing LDH-phosphate (Figure 4b) and LDH-phosphinate (Figure 4d) displayed single LDH layers (exfoliated structure) homogenously dispersed in the PBAT matrix although a few agglomerates of non-exfoliated LDH layers could also be observed, especially in the latter case. In contrast, the PBAT nanocomposite with LDH-decanoate (Figure 4c) showed the formation of LDH-rich domains in the PBAT matrix. The LDH-decanoate filler was swelled in monomers and delaminated during the in-situ polymerization (as evidenced from the XRD patterns—Figure 5) but the formed LDH sheets covered with IL-decanoate anions showed a low tendency to migrate into the surrounding PBAT matrix probably due to limited PBAT/IL-decanoate miscibility. It is known that a few % addition of phosphonium ILs into PBAT leads to a phase separation and formation of ionic clusters [41].

The XRD patterns of PBAT nanocomposites clearly confirmed the morphologies observed by TEM. The XRD pattern of PBAT nanocomposite with pristine LDH (Figure 5b) shows the intensive (003) diffraction peak at 2*θ* = 11.8° (slightly shifted to higher angles due to a lower content of interlayer water in freshly dried LDH) revealing that the layered particles were neither exfoliated nor intercalated during the in-situ polycondensation. On the contrary, a complete disappearance of the reflection peak at a low angle range (2*θ* < 4°) was observed for all PBAT nanocomposites with the modified LDH (Figure 5c–e) suggesting extensive exfoliation of the IL-anion-intercalated LDH particles. Moreover, in the cases of PBAT nanocomposites with the modified LDH, the XRD peak attributed to the second phase of LDH with smaller basal spacing, originally present at 2*θ* = 10.77–11.45° (see Figure 2) was significantly decreased, broadened, and/or even shifted to lower angles (Figure 5c–e). It indicates that this LDH fraction was also partially delaminated and intercalated by PBAT chain during the in-situ polycondensation in the presence of IL-anion modified fillers.

The crystalline reflections associated with the PBAT matrix were observed at higher 2*θ* angles of 16.2°, 17.5°, 20.5°, 23.2°, and 24.9° (Figure 5) relating to the characteristic (0̅11), (010), (1̅11), (100), and (1̅11) planes, respectively [5,26]. The XRD data for all samples were identical in this region confirming that the LDH additions up to 5 wt % had not affected the crystalline structure of the PBAT matrix [26].

#### 3.2.2. Water Vapor and Gas Barrier Properties

The transport properties of O_2_, CO_2_ gases, and water vapor in the neat PBAT and the PBAT nanocomposites were investigated and permeability (Table 2), diffusion (Table S1), and solubility (Table S2) coefficients, as well as their ideal selectivities were determined.

The permeability coefficients of all samples increase in the order of O_2_ < CO_2_ << H_2_O (Table 2). The permeability measurements were performed at temperatures at which the PBAT nanocomposites were in a rubbery state (above *T*_g_—see the DSC result below). In such conditions, the permeation, diffusion, and solubility mechanisms in the system resembled gas transport in liquids. Crystallinity of the neat PBAT film (8%) was slightly enhanced by the presence of LDH fillers (<13%—see the DSC result below). However, such low overall crystallinity in the PBAT/LDH nanocomposites has a negligible effect on the evolution of gas transport properties [10].

Since the permeability of gas and water vapor molecules was directly proportional to diffusion and solubility coefficients, the more dominating process can be determined based on the diffusion and solubility selectivities. In the cases of neat PBAT and PBAT/LDH nanocomposites, the gas and water vapor permeabilities were preferably driven by solubility in PBAT rather than by diffusion (the values of solubility selectivities were much higher than the diffusion ones) (Tables S1 and S2). Generally, solubility is correlated with the amount and intensity of interactions between the penetrant and polymer matrix [4,42,43].

It seems that the relatively high polarity of the PBAT backbone caused by the presence of ester groups promotes strong interactions between PBAT and polar molecules such as CO_2_ and H_2_O [2]. As a result, the determined solubility coefficients of CO_2_ and H_2_O are much higher than those of O_2_, which further results in significantly faster permeation of these polar molecules through PBAT materials as compared to O_2_. Neutral oxygen interacted weakly with PBAT and therefore relatively high selectivities for CO_2_/O_2_ gases were obtained.

The LDH fillers in PBAT nanocomposites were shown to slightly lower CO_2_ and O_2_ permeations by acting as a physical barrier, decreasing diffusion but only negligibly affecting solubility, which is in agreement with the theory of transport properties of polymeric materials filled with impermeable particles [44,45]. Gas molecules penetrate through free volume among the PBAT chains and the presence of LDH particles increases tortuosity of the gas molecule pathways. In our case, creation of longer diffusion paths for gas molecules depended mainly on the uniform dispersion and the aspect ratio of the 2D nanoplatelets. In the cases of nanocomposites with the modified LDH, the intercalated IL-anions promoted the LDH delamination, giving an abundance of homogenously dispersed LDH nanoplatelets throughout the PBAT matrix (Figure 4). This then contributed to the decreased values of diffusion coefficients at 5 wt % LDH (Table S1). Below this filler content, the effect of LDH addition and delamination was negligible due to a relatively low aspect ratio of the produced nanoplatelets. Unfortunately, the one-pot co-precipitation method of LDH synthesis in the presence of ILs did not allow for the formation of LDH with high lateral dimension as shown for materials synthesized by anion-exchange reaction, published in our previous study [8].

**Figure 6:**
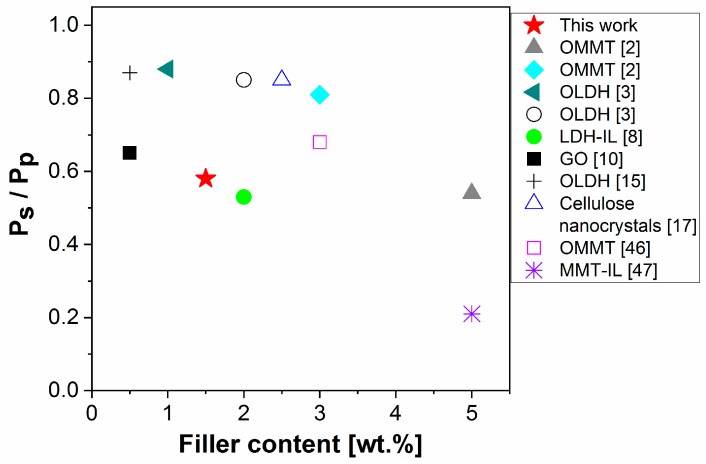
Comparison of relative permeability values (P_s_/P_p_) between this work (PBAT nanocomposite with 1.5 wt % of LDH-phosphate) and other PBAT/filler nanocomposites.

Contrary to CO_2_ and O_2_ transport properties, the neat PBAT exhibited high water vapor permeabilities (WVP, Table 2), which limits its use for food packaging [2,3]. The addition of pristine LDH resulted in a very slight WVP decrease (6.5%) due to poor filler dispersion in the PBAT matrix (Figure 4a). In contrast, the incorporation of modified LDH into the PBAT matrix led to significant WVP decreases varying in function of the chemical nature of the IL-anions. The most significant decrease in WVP coefficient was observed for the PBAT nanocomposite with 5.0 wt % of LDH-decanoate (44% reduction). However, it is of great importance to improve the water vapor barrier properties of PBAT at low nanofiller loadings to avoid viscosity increase and limited processability for food packaging films fabrication [3]. From the point of view of this application, the PBAT nanocomposite with 1.5 wt % of LDH-phosphate, in which WVP was reduced by 46% (Table 2) was selected as the most promising and used for comparison with other PBAT/filler nanocomposites with similar nanofiller loadings. Relative permeabilities (P_s_/P_p_), where P_s_ and P_p_ are WVP of PBAT/filler (in our case PBAT/LDH) nanocomposite and neat PBAT, respectively, are depicted in Figure 6.

Our results are promising compared to the results so far published in the literature. They show the most significant improvement in the water vapor barrier properties of PBAT/LDH-phosphate nanocomposites, especially considering the relatively low nanofiller content (1.5 wt %). The materials prepared in our last study displayed similar WVP results only when higher amounts (2 wt %) of IL-modified LDH was incorporated into the PBAT matrix [8]. In that case, the PBAT nanocomposites exhibited partially exfoliated/intercalated morphology since the LDH delamination occurred to a minor extent due to the low content of intercalated IL. It seems that the vapor barrier properties of PBAT/LDH/IL systems are driven by the dispersion and exfoliation degree of LDH particles as well as by the formation of ionic clusters [8]. Highly polar surface (OH groups) of well dispersed LDH particles create more sorption sites (increased interactions) for polar water molecules which can more feasibly form clusters in the permeation pathways causing a significant decrease of water diffusion and thus overall decreased water permeation through the nanocomposites.

Other lamellar fillers (e.g., the most frequently used organically modified montmorillonite—OMMT, [2,3,46]) have been shown to provide similar WVP decrease of the PBAT nanocomposites at much higher OMMT loadings (e.g., 5 wt % [2], Figure 6). However, when non-polar phosphonium IL-modified montmorillonite is introduced, the relative permeability values of the PBAT/OMMT nanocomposites can be decreased even further (down to 0.2, Figure 6) [47].

#### 3.2.3. Thermal Properties

Table 3 summarizes the results obtained by DSC. The glass transition temperature (*T_g_*) values increased with the increasing content of the modified LDH containing IL-anions, which indicates homogenous nanofiller dispersion in the PBAT matrix and LDH delamination into individual LDH layers thus reducing mobility of PBAT chains. In contrast, the incorporation of pristine LDH showed no influence on *T_g_* due to poor nanofiller dispersion in the PBAT matrix. The increase in crystallinity (*X_c_*) and the decrease in melting temperature (*T_m_*) of PBAT with increasing content of modified LDH indicates that the particles act as heterogeneous nucleating agents promoting the PBAT crystallite growth [8,9,15].

The TGA results show that all prepared materials were thermally stable up to ca 280 °C. The LDH incorporation had slightly decreased the thermal stability (*T_d_*_5%_) of the PBAT matrix (Table 3). This effect was more significant for the modified LDH with IL-anions and thus probably connected to the degradation of IL-anions and possible catalytic effect of IL-anions on thermal degradation of PBAT at elevated temperature [15].

#### 3.2.4. Mechanical Properties

Table 4 summarizes the results of uniaxial tensile properties of the prepared PBAT nanocomposite films. The LDH addition has led to a general improvement in stiffness (the increased Young moduli) as a consequence of increased rigidity induced by the nanofiller incorporation into the PBAT matrix and a slightly higher amount of the crystalline phase. However, tensile strength values have improved only in the PBAT/LDH nanocomposites with IL-phosphate and IL-phosphinate modifications. It means that these two modifications provided the most homogeneous LDH dispersion within the PBAT matrix. The best affinity between the LDH filler and PBAT was provided by IL-phosphinate as shown by the improved values of elongation at break. As we demonstrated in our last study, the IL-phosphinate modifier promotes formation of well-dispersed ionic clusters in PBAT, which can be responsible for increased ductility without reducing stiffness of the material [8,48]. In contrast, the decreased values of tensile strength and elongation at break of the PBAT nanocomposites containing LDH modified with IL-decanoate give evidence of material brittleness originating probably from the phase-separated morphology with LDH-rich domains in the PBAT matrix (see Figure 4). The brittle behavior was also observed for the PBAT nanocomposites with non-modified LDH as a result of poor nanofiller dispersion and a presence of large LDH agglomerates.

## 4. Conclusions

In this work, the preparation of PBAT/LDH nanocomposites by in-situ polycondensation in the presence of IL-anion modified LDH was reported.

First, a direct co-precipitation of metal salts in the presence of phosphonium ionic liquids (IL) was used for synthesis of IL-anion intercalated LDH. Using this technique, bis (2-ethylhexyl) phosphate (IL-phosphate), decanoate (IL-decanoate), and bis (2,4,4-trimethylpentyl) phosphinate (IL-phosphinate) anions were successfully introduced into the interlayer space of LDH compounds. The produced LDH show the increased interlayer spacing and high content of IL-anions. The results surprisingly show that LDH-IL prepared by this technique practically does not contain surface-bonded IL (in contrast to anion exchange technique used in our last study [8]). The way in how to prepare LDH containing both intercalated IL-anions and surface bonded IL is still challenging and under our investigation.

In the next step, LDH with intercalated IL-anions were shown to delaminate readily in a mixture of monomers during in-situ polycondensation. The produced PBAT/LDH nanocomposites exhibited exfoliated morphology either with homogenously dispersed LDH nanoplatelets in the PBAT matrix (the cases of IL-phosphate and IL-phosphinate intercalated LDH) or formation of LDH-rich domains in the PBAT matrix (the case of IL-decanoate intercalated LDH). Moreover, the IL-phosphinate modifier was shown to ensure the strongest LDH-PBAT affinity resulting in optimized mechanical performances. The presence of IL-anion intercalated LDH exhibited a relatively low effect on CO_2_ and O_2_ permeability reduction while the water vapor permeation was significantly decreased for all PBAT/IL-modified LDH nanocomposites. From this point of view, the produced PBAT/LDH nanocomposites are considered excellent candidates for food packaging applications.

## Figures and Tables

**Table 1:** Ionic liquids used for the modification of layered double hydroxides (LDH).

Ionic Liquid	Chemical Structure	Designation
Trihexyltetradecylphosphonium bis (2,4,4-trimethylpentyl) phosphinate	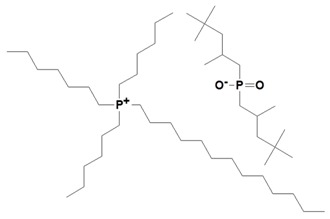	IL-phosphinate
Trihexyltetradecylphosphonium decanoate	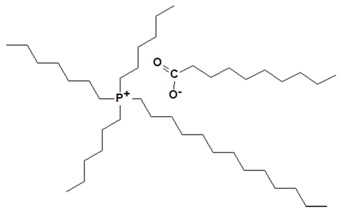	IL-decanoate
Trihexyltetradecylphosphonium bis (2-ethylhexyl) phosphate	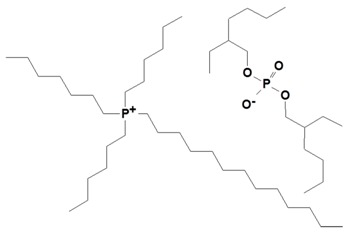	IL-phosphate

**Table 2:** Comparison of O_2_, CO_2_, and water vapor permeabilities and ideal selectivities of neat PBAT and PBAT nanocomposites with 1.5 and 5 wt % of pristine LDH, LDH-phosphate, LDH-decanoate, and LDH-phosphinate.

	Permeability Coefficient (Barrer) ^1^			Ideal Selectivity		
	O_2_	CO_2_	H_2_O	CO_2_/O_2_	H_2_O/O_2_	H_2_O/CO_2_
Neat PBAT	1.55	16.9	2730	10.9	1770	162
+1.5% pristine LDH	1.42	15.5	2660	10.9	1880	172
+1.5% LDH-phosphate	1.35	14.3	1570	10.6	1170	110
+1.5% LDH-decanoate	1.48	16.1	2010	10.9	1358	125
+1.5% LDH-phosphinate	1.48	16.3	1980	11.0	1338	121
+5% pristine LDH	1.33	15.1	2550	11.4	1930	169
+5% LDH-phosphate	1.30	14.1	2300	10.8	1770	163
+5% LDH-decanoate	1.21	12.5	1530	10.3	1264	122
+5% LDH-phosphinate	1.43	14.0	1740	9.8	1217	124

^1^ Barrer = 1 × 10^−10^ cm^3^ (STP) cm·cm^−2^·s^−1^ cmHg^−1^ = 3.3539 × 10^−16^ mol·s^−1^·m^−1^·Pa^−1^.

**Table 3:** DSC and TGA results of neat PBAT and PBAT nanocomposites with 1.5 and 5 wt % of pristine LDH, LDH-phosphate, LDH-decanoate, and LDH-phosphinate.

	*T_g_* [°C]	*T_m_* [°C]	Δ*H_m_* [J/g]	*X_c_* [%]	*T_d_*_5%_ [°C]
Neat PBAT	−40	116	9.7	8	354
+1.5% pristine LDH	−39	108	11.8	11	351
+1.5% LDH-phosphate	−36	111	10.1	9	336
+1.5% LDH-decanoate	−38	103	11.4	10	331
+1.5% LDH-phosphinate	−38	110	10.9	10	342
+5% pristine LDH	−40	113	8.3	8	346
+5% LDH-phosphate	−37	102	11.3	10	317
+5% LDH-decanoate	−37	99	13.6	13	310
+5% LDH-phosphinate	−35	101	13.2	12	324

**Table 4:** Tensile properties of neat PBAT and PBAT nanocomposites with 1.5 and 5 wt % of pristine LDH, LDH-phosphate, LDH-decanoate, and LDH-phosphinate.

	Young Modulus [MPa]	Tensile Strength [MPa]	Elongation at Break [%]
Neat PBAT	76 ± 4	5.6 ± 0.3	122 ± 23
+1.5% pristine LDH	92 ± 4	5.9 ± 0.4	121 ± 18
+1.5% LDH-phosphate	110 ± 2	6.8 ± 0.4	133 ± 20
+1.5% LDH-decanoate	104 ± 4	5.0 ± 0.2	13 ± 3
+1.5% LDH-phosphinate	94 ± 2	8.7 ± 0.5	227 ± 15
+5% pristine LDH	99 ± 4	5.2 ± 0.5	29 ± 16
+5% LDH-phosphate	106 ± 4	6.2 ± 0.2	20 ± 3
+5% LDH-decanoate	137 ± 4	5.1 ± 0.1	10 ± 1
+5% LDH-phosphinate	112 ± 5	8.3 ± 0.6	225 ± 43

## Supplementary Materials

The following are available online at https://www.mdpi.com/2079-4991/9/4/618/s1, Figure S1: ^1^H NMR spectrum of synthesized PBAT, Figure S2: FTIR spectra of (a) IL-phosphate, (b) IL-decanoate and (c) IL-phosphinate, Table S1: Dependence of oxygen, carbon dioxide and water vapor diffusion coefficient and ideal selectivity of neat PBAT and PBAT nanocomposites with 1.5 and 5 wt % of pristine LDH, LDH-phosphate, LDH-decanoate and LDH-phosphinate, Table S2: Dependence of oxygen, carbon dioxide and water vapor solubility coefficient and ideal selectivity of neat PBAT and PBAT nanocomposites with 1.5 and 5 wt % of pristine LDH, LDH-phosphate, LDH-decanoate and LDH-phosphinate.
