# Supplementary material for: Ionic Liquids as Delaminating Agents of Layered Double Hydroxide during In-Situ Synthesis of Poly (Butylene Adipate-co-Terephthalate) Nanocomposites
Source: Nanomaterials (Basel). 2019 Apr 16;9(4):618. doi: 10.3390/nano9040618 (PMC6523161; doi:10.3390/nano9040618)
Supplement: Supplementary file 1 [file nanomaterials-09-00618-s001.pdf]

Supporting information

Ionic Liquids as Delaminating Agents of Layered Double Hydroxide during In-Situ Synthesis of Poly (Butylene Adipate-*co*-Terephthalate) Nanocomposites

Hynek Beneš [1,*], Jana Kredatusová [1], Jakub Peter [1], Sébastien Livi [2], Sonia Bujok [1], Ewa Pavlova [1], Jiří Hodan [1], Sabina Abbrent [1], Magdalena Konefał [1] and Petra Ecorchard [3]

[1] Institute of Macromolecular Chemistry of the Czech Academy of Sciences, Heyrovského nám. 2, 162 06 Prague 6, Czech Republic; jana.kredatusova@email.cz (J.K.); peter@imc.cas.cz (J.P.); bujok@imc.cas.cz (S.B.); pavlova@imc.cas.cz (E.P.); hodan@imc.cas.cz (J.H.); abbrent@imc.cas.cz (S.A.); magdalenakonefal@imc.cas.cz (M.K.)

[2] Université de Lyon, CNRS, UMR 5223, Ingénierie des Matériaux Polymères, INSA Lyon, F-69621 Villeurbanne, France; sebastien.livi@insa-lyon.fr

[3] Institute of Inorganic Chemistry of the Czech Academy of Sciences, Husinec-Řež č.p. 1001, 25068 Řež, Czech Republic; ecorchard@iic.cas.cz

* Correspondence: benesh@imc.cas.cz; Tel.: +420-296-809-313

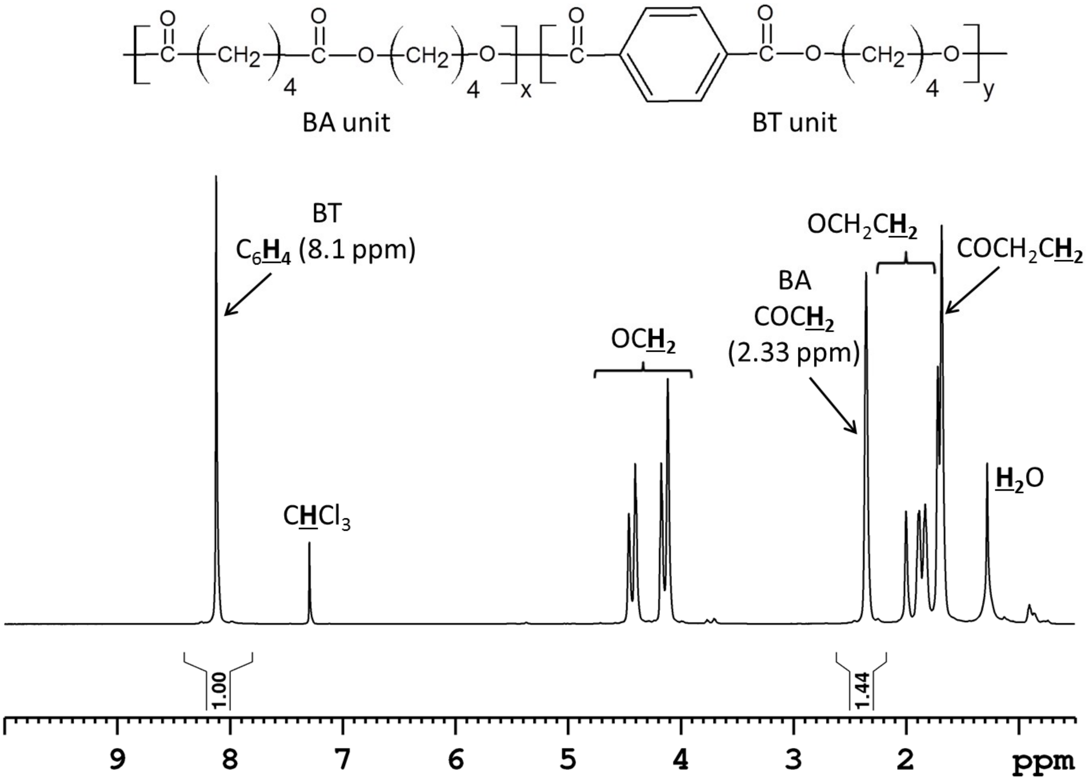

Figure S1: ^{1}H NMR spectrum of synthesized poly(butylene adipate-co-terephthalate)

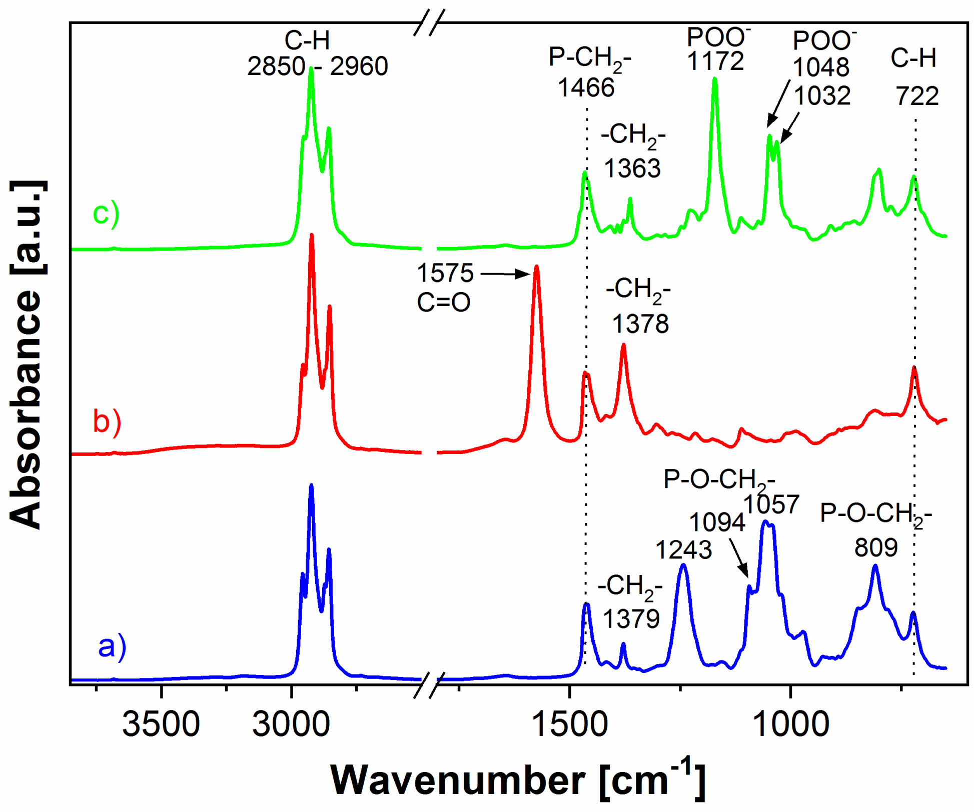

Figure S2: FTIR spectra of a) IL-phosphate, b) IL-decanoate and c) IL-phosphinate

Table S1: Dependence of oxygen, carbon dioxide and water vapor diffusion coefficient and ideal selectivity of neat PBAT and PBAT nanocomposites with 1.5 and 5 wt.% of pristine LDH, LDH-phosphate, LDH-decanoate and LDH-phosphinate

	Diffusion coefficient $\times 10^{12}$ m^2/s			Diffusion selectivity		
	O_2	CO_2	H_2O	CO_2/O_2	H_2O/O_2	H_2O/CO_2
Neat PBAT	34.2	13.1	15.6	0.38	0,46	1.10
+1.5% LDH	32.8	12.9	14.9	0.39	0.45	1.10
+1.5% LDH-phosphate	29.8	11.8	11.7	0.40	0.39	1.00
+1.5% LDH-decanoate	25.2	14.6	12.2	0.58	0.48	0.83
+1.5% LDH-phosphinate	30.9	15.1	13.8	0.49	0.45	0.91
+5% LDH	31.3	12.7	13.0	0.41	0.41	1.20
+5% LDH-phosphate	26.5	11.0	8.7	0.42	0.33	0.79
+5% LDH-decanoate	27.6	11.0	7.4	0.40	0.27	0.68
+5% LDH-phosphinate	28.5	13.0	9.9	0.46	0.35	0.76

Table S2: Dependence of oxygen, carbon dioxide and water vapor solubility coefficient and ideal selectivity of neat PBAT and PBAT nanocomposites with 1.5 and 5 wt.% of pristine LDH, LDH-phosphate, LDH-decanoate and LDH-phosphinate

	Solubility coefficient $\times 10^7$ $mol/(m^3.Pa)$			Solubility selectivity		
	O_2	CO_2	H_2O	CO_2/O_2	H_2O/O_2	H_2O/CO_2
Neat PBAT	1.52	43.4	5900	28.5	3880	140
+1.5% LDH	1.46	40.4	6000	27.8	4120	150
+1.5% LDH-phosphate	1.52	40.8	4500	26.9	2970	120
+1.5% LDH-decanoate	1.98	36.9	5600	18.7	2840	160
+1.5% LDH-phosphinate	1.61	36.3	4900	22.6	3050	140
+5% LDH	1.42	39.9	6600	28.1	4650	170
+5% LDH-phosphate	1.65	42.9	8900	25.9	5380	210
+5% LDH-decanoate	1.47	38.4	7000	26.1	4770	190
+5% LDH-phosphinate	1.69	36.3	6000	21.5	3550	170
